# Establishing Minimally Invasive Cardiac Surgery in a Developing Country: A Five-Year Experience at Hayatabad Medical Complex, Pakistan

**DOI:** 10.7759/cureus.74659

**Published:** 2024-11-28

**Authors:** Muhammad Aasim, Raheela Aziz, Atta ul Mohsin, Raheel Khan, Ayesha Zahid, Jibran Ikram

**Affiliations:** 1 Cardiac Surgery, Hayatabad Medical Complex Peshawar, Peshawar, PAK; 2 Cardiovascular Medicine, Hayatabad Medical Complex Peshawar, Peshawar, PAK

**Keywords:** minimal invasive cardiac surgery, minimal invasive direct coronary artery bypass, mini-sternotomy, surgical outcomes, thoracotomy

## Abstract

Background

The adoption of minimally invasive cardiac surgery (MICS) has increased over the past 25 to 30 years, driven by advancements in technology and a growing understanding of its benefits. This study evaluates the outcomes of 144 elective MICS procedures performed between January 2019 and September 2024.

Methods

Patients underwent various surgical approaches, including upper mini-sternotomy, mini-thoracotomy, and sub-xiphoid access. Patient demographics, preoperative characteristics, and surgical outcomes were analyzed. A total of 144 MICS procedures were performed.

Results

The cohort had a mean age of 30.72 years, with a nearly equal gender distribution, indicating that MICS can be performed safely in Pakistani patients. The mean ejection fraction was 53.58%, with hypertension being the most common comorbidity (22.2%). Aortic cannulation was primarily utilized, and aortic valve replacement (AVR) was the most common procedure (44.4%). The mean cardiopulmonary bypass (CPB) time was 95.9 ± 56.3 minutes and the mean aortic cross-clamp time was 62.22 ± 57.004 minutes, demonstrating efficient procedural times. The overall incidence of complications was low, supporting the safety and efficacy of MICS.

Conclusion

Our findings suggest that MICS is a viable and effective approach for a diverse patient population, with favorable clinical outcomes. The results underscore the potential for MICS to become standard practice in cardiothoracic surgery. Future research should focus on long-term outcomes and the influence of comorbidities to further enhance MICS methodologies.

## Introduction

Minimally invasive cardiac surgery (MICS) is defined as any cardiac surgery done without full median sternotomy, or thoracotomy incision less than 12 cm in length, irrespective of whether on or off cardiopulmonary bypass (CPB) [1]. Over the last two decades, MICS has evolved into a well-established technique for the treatment of cardiac surgical conditions [2-6]. The growing popularity of MICS is largely due to significant advancements in technology, coupled with a rising demand from both surgeons and patients for less invasive methods to treat cardiac conditions that require surgical solutions [7,8]. It wasn't until the mid-1990s that minimally invasive approaches were introduced in cardiac surgery [9]. A range of alternative access techniques has been described in the literature, including partial sternotomy, limited-access thoracotomy, fully endoscopic procedures, catheter-based hybrid approaches, and methods utilizing sub-xiphoid and subdiaphragmatic access [10-12]. In MICS, smaller incisions lower the risk of wound infections associated with sternal trauma and prevent complications related to sternum healing [13]. Additional potential advantages of MICS include a decrease in postoperative atrial fibrillation [14]. Other benefits include shorter hospital stays [15], faster patient mobilization [16], and enhanced cost-effectiveness relative to standard on-pump coronary artery bypass grafting (CABG) [15]. As MICS becomes more accepted and established in cardiac surgery, it is essential to continually assess outcomes to confirm that the smaller incision does not compromise surgical results and to support effective patient selection. This study reviews five-year experience with MICS at a single institute.

## Materials and methods

From January 1, 2019, to September 30, 2024, a total of 144 MICS procedures were performed. In this study, MICS is defined in accordance with the New York State reporting guidelines as any cardiac surgical operation conducted through an incision other than a full median sternotomy. Data were collected from the patients’ files in an Excel sheet of a computer on demographics, surgical parameters, and short-term morbidity and mortality outcomes. The main outcomes of interest comprised conversion to full median sternotomy, conversion from off-bypass to on-bypass, duration of cross-clamping, CPB time, length of ICU and hospital stay, and mortality rates. Patients with severe calcification or aortic pathology unsuitable for MICS were excluded. Continuous variables were expressed as mean ± standard deviation, while categorical variables were indicated as percentages. Cases without an aortic cross-clamp and those performed without CPB were not included in the calculations for mean cross-clamp and CPB times, respectively.

MICS incisions in our practice

The MICS in our practice included in this study are as follows.

Upper Mini-Sternotomy (UMS)

**Figure 1:**
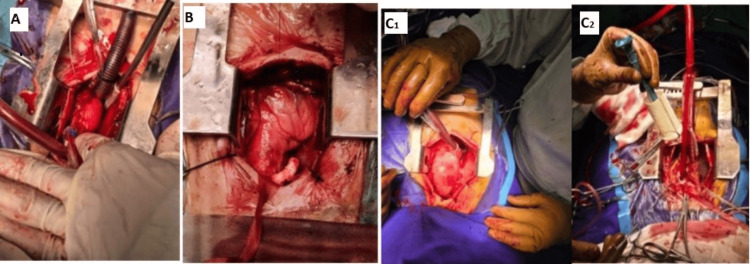
Upper mini-sternotomy A: Shows upper mini-sternotomy for aortic valve replacement and aortic root enlargement. B: Shows upper mini-sternotomy for modified central shunt (Aasim’s shunt) in adult congenital heart disease with hypoplastic pulmonary valve severe stenosis and pulmonary vessels. C1 and C2: Shows upper mini-sternotomy for modified Bentall aortic valve replacement surgery as standard treatment for type A aortic dissection.

We use this surgical incision access primarily for aortic valve replacement (AVR), sub-aortic membrane resection, aortic root enlargement, aortic valvuloplasty, modified central shunt (Aasim’s shunt), subaortic left ventricular myomectomy, and mini-Bentall procedures involving aortic root replacement (Figure 1).

Left-Side Thoracotomy

**Figure 2:**
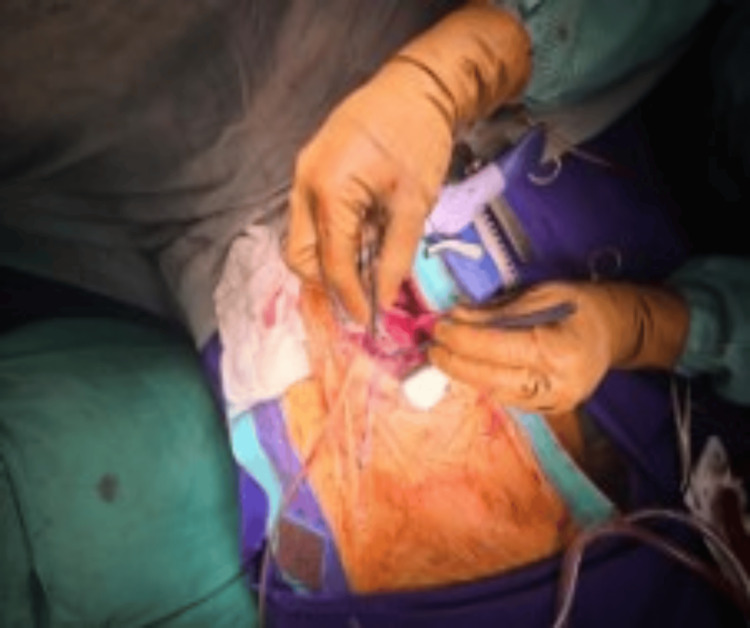
Left thoracotomy approach for MIDCAB MIDCAB, minimally invasive direct coronary artery bypass.

We commonly use this access for minimally invasive direct coronary artery bypass (MIDCAB), repair of coarctation of the aorta, pericardiopleural windows, pulmonary artery banding, redo epicardial permanent pacemaker (PPM) implantation, and ligation of patent ductus arteriosus (PDA) (Figure 2).

Right-Side Thoracotomy

**Figure 3:**
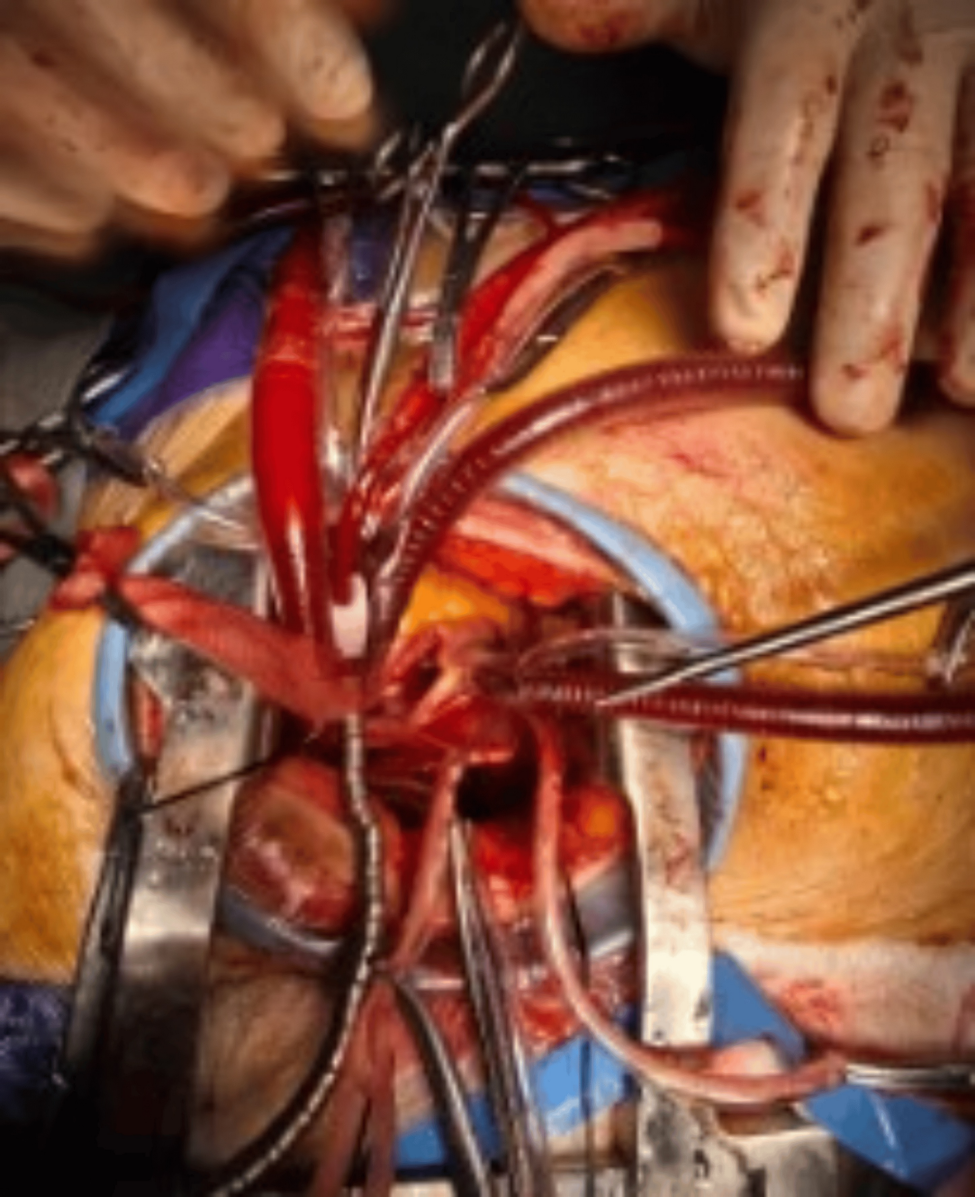
Right mini-thoracotomy MICS for ASD-II closure, showing arrangements of the cardiopulmonary bypass cannulae and use of Cosgrove aortic cross-clamp MICS, minimally invasive cardiac surgery; ASD-II, secundum atrial septal defect.

We typically use this type of MICS incision for atrial septal defect (ASD) closure and interventions on the mitral and tricuspid valves (Figure 3).

Sub-Xiphoid Incision

We use this MICS surgical approach mainly for epicardial PPM implantation, pericardial biopsy, and surgical pericardial drainage.

## Results

A total of 144 patients underwent elective MICS from January 2019 to September 2024. Baseline demographic details are outlined in Table 1.

**Table 1:** Demographic data CVA, cerebrovascular accident; EF, ejection fraction.

Parameter	Value
Age, mean ± SD	30.72 ± 18.634
Sex	
Male	74 (51.4%)
Female	70 (48.6%)
Diabetic	18 (12.5%)
Smoker	12 (8.3%)
Hypertension	32 (22.2%)
Renal failure	4 (2.8%)
Endocarditis	7 (4.9%)
CVA	3 (2.1%)
EF%, mean ± SD	53.58 ± 6.929

The mean age of the participants was 30.72 ± 18.634 years, with a near-equal gender distribution: 51.4% men and 48.6% women. The average ejection fraction among the patients was 53.58 ± 6.929. Various preoperative conditions were observed, with hypertension being the most prevalent, affecting 22.2% of the group. Additionally, 2.8% of patients had a history of renal failure, requiring dialysis or kidney transplantation. Prior cerebrovascular accidents were present in 2.1% of the cohort, while 4.9% had been treated for endocarditis.

**Figure 4:**
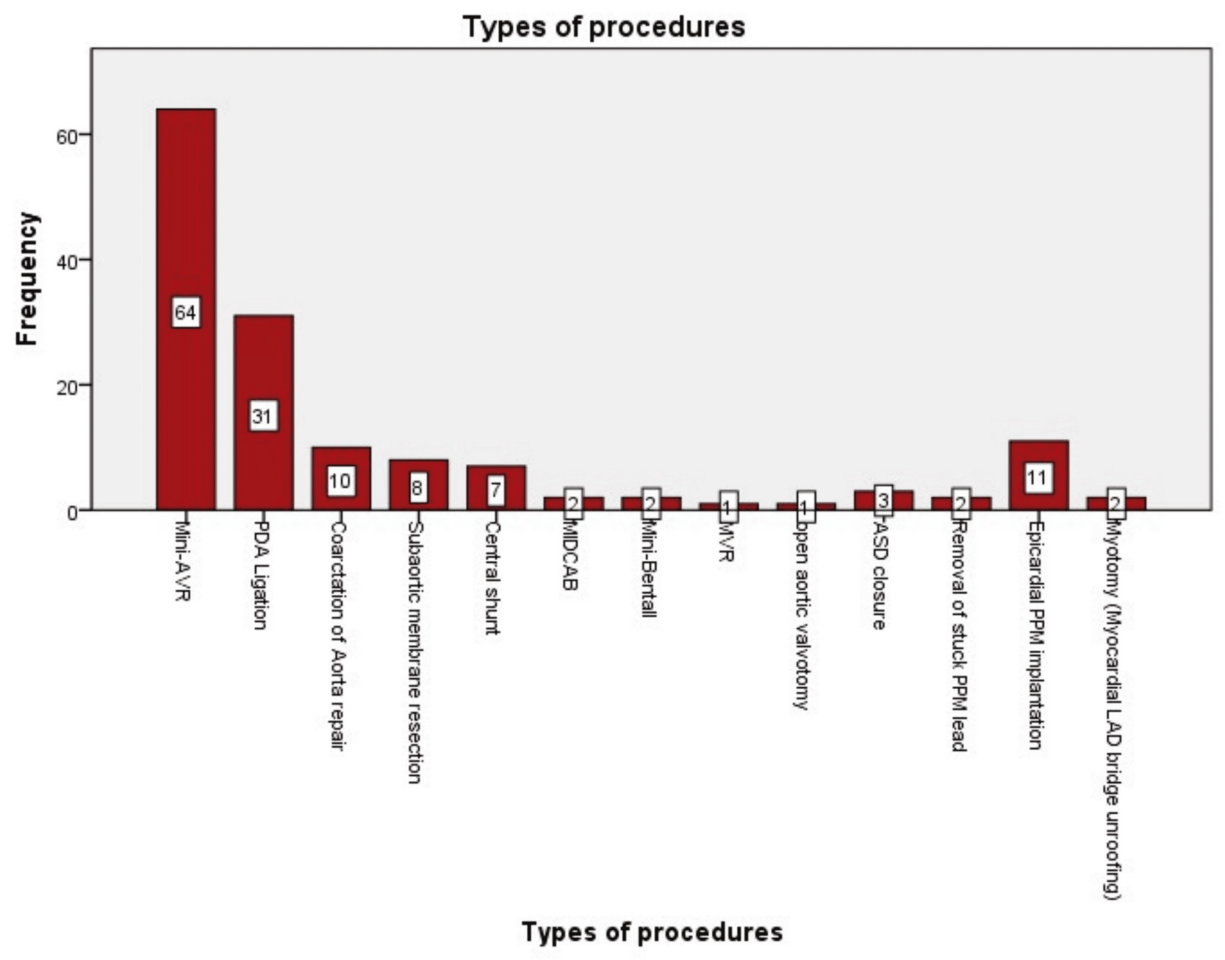
Illustration of distribution of cases LAD, left anterior descending; PPM, permanent pacemaker; ASD, atrial septal defect; PDA, patent ductus arteriosus; AVR, aortic valve replacement; MIDCAB, minimally invasive direct coronary artery bypass; MVR: mitral valve replacement.

The four most commonly performed procedures included AVR at 44.4% (n = 64), patent ductus arteriosus (PDA) ligation at 21.5% (n = 31), epicardial pacemaker implantation at 7.6% (n = 11), and coarctation of the aorta repair at 6.9% (n = 10). Additional procedures performed included CABG at 1.4% (n = 2), ASD repair at 2.1% (n = 3), mitral valve replacement at 0.7% (n = 1), myotomy (myocardial LAD (left anterior descending) bridge unroofing) at 1.4% (n = 2), removal of a stuck pacemaker lead at 1.4% (n = 2), subaortic membrane resection at 5.6% (n = 8), central shunt at 4.9% (n = 7), and open aortic valvotomy at 0.7% (n = 1). Notably, beginning in August, we also commenced the mini-Bentall procedure, which accounted for 1.4% of the cases (n = 2). The distribution of cases is illustrated in Figure 4.

**Figure 5:**
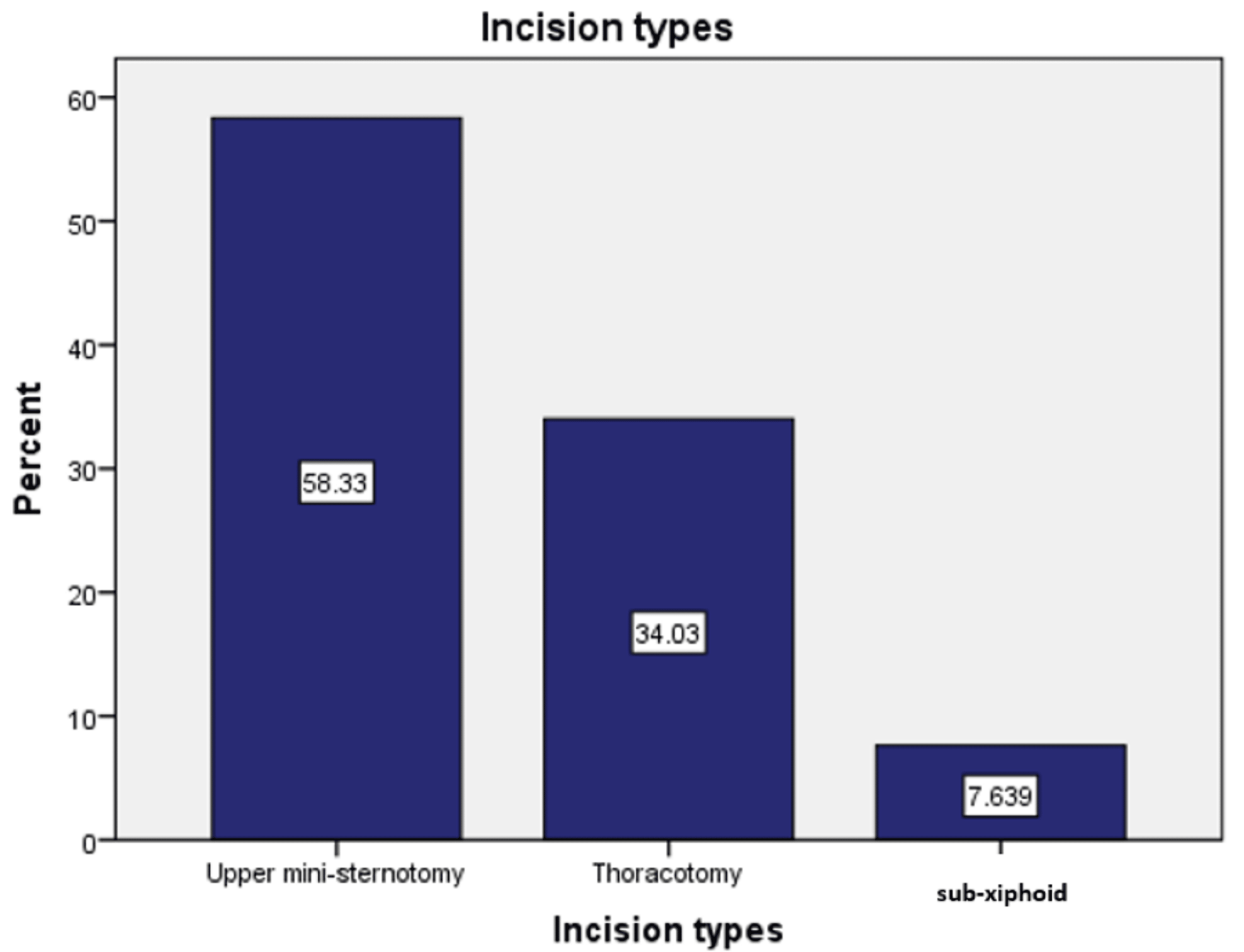
Types of incisions and their percentages

The majority of procedures were performed using a UMS (58.33%), followed by mini-thoracotomy (34.03%) and sub-xiphoid incision (7.64%). UMS was primarily utilized for AVR and mini-Bentall procedures involving aortic root replacement. Left-side thoracotomy was commonly used for MIDCAB, repair of coarctation of the aorta, and ligation of PDA. Right-side thoracotomy was typically performed for ASD closure, as well as interventions on the mitral and tricuspid valves. The sub-xiphoid approach was mainly used for epicardial PPM implantation, although redo cases occasionally required a left mini-thoracotomy. The distribution of incision types is presented in Figure 5.

**Figure 6:**
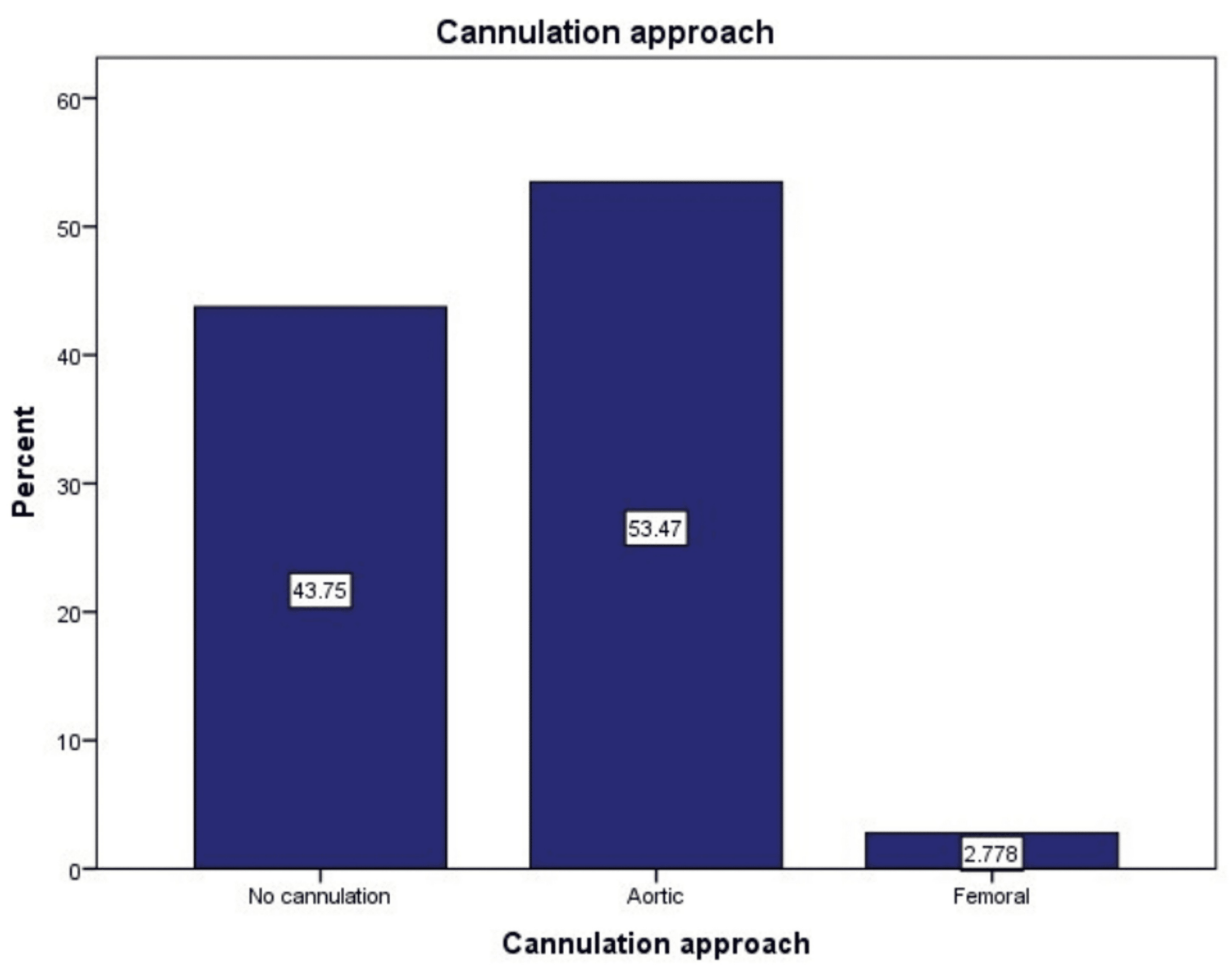
The distribution of cannulation techniques, highlighting that aortic cannulation (53.7%) was the most frequently employed, followed by femoral cannulation (2.7%) The cases labeled as "no cannulation" (43.75%) in the graph represent procedures performed off bypass.

The choice of cannulation methods highlights that aortic cannulation (53.7%) was the most frequently employed, followed by femoral cannulation (2.7%). The cases labeled as "no cannulation" (43.75%) represent procedures performed off bypass (Figure 6).

Across the entire series, the mean aortic cross-clamp time was 62.22 ± 57.004 minutes, while the mean CPB time was 95.9 ± 56.3 minutes. There were no conversions to full median sternotomy or transitions from off bypass to on bypass. The average length of hospital stay was 5.06 ± 0.446 days, while the average ICU stay was 1.92 ± 0.365 days. The in-hospital mortality rate was 0%, and the incidence of sternal wound infection was 1.4%.

## Discussion

Although minimally invasive techniques have long been established in various surgical disciplines, the significant adoption of MICS has primarily occurred in the past 25 to 30 years. Early hesitation in embracing these methods in cardiac surgery stemmed from worries about intracardiac air, restricted access to major procedures, and challenges with cannulation. However, since the mid-1990s, technological advancements have accelerated the integration of MICS for a range of cardiac operations. Consequently, in certain specialized centers, MICS has become the standard practice for heart surgery [1]. Our study evaluated the outcomes of 144 elective MICS performed between January 2019 and September 2024, utilizing various surgical approaches, including UMS, mini-thoracotomy, and sub-xiphoid access. The diversity in approaches reflects our commitment to tailoring surgical techniques to individual patient needs, enhancing recovery times, and minimizing complications.

The choice of cannulation methods - primarily aortic cannulation (more commonly utilized than femoral) - was aligned with established practices that prioritize access and minimize complications. Aortic dissection is a rare complication, occurring in 0.01% to 0.09% of patients undergoing central aortic cannulation [17]. Signs such as a bluish hue of the aorta or an elevated arterial line pressure after initiating CPB should trigger a heightened suspicion of dissection. Prompt recognition is crucial, as timely intervention can significantly impact patient outcomes [18].

The diverse surgical approaches, including mini-sternotomy and mini-thoracotomy, allowed us to effectively manage various pathologies while maintaining a focus on minimally invasive techniques. UMS is mainly used for AVR and Bental aortic root replacement procedures and is most suitable for central cannulation [19-21,22]. Left-side thoracotomy is mainly used for MIDCAB, coarctation of aorta repair, and PDA ligation. Right-side thoracotomy is mainly used for mitral valve [23-26] and tricuspid valve interventions and ASD closure. The sub-xiphoid approach is mainly used for epicardial PPM implantation and sometimes may require left mini-thoracotomy (especially in redo cases). The most common procedure was AVR, performed in 44.4% of cases. These findings underscore the significance of aortic valve pathology in our practice, as well as the utility of MICS in addressing congenital heart conditions like PDA. The successful implementation of the mini-Bentall procedure [27,28] in 1.4% of cases demonstrates our evolving skill set and commitment to expanding the indications for minimally invasive techniques.

This study has some limitations that should be acknowledged. First, as a single-center experience, the findings may not be generalizable to other institutions, particularly in different geographic or resource settings. Second, the sample size, while robust for an initial analysis, limits our ability to perform subgroup analyses for specific comorbidities or individual procedural techniques. Additionally, we only assessed short-term outcomes, leaving long-term impacts of MICS, such as survival rates, quality of life, and durability of procedures, unexplored. Further multicenter studies are needed on this topic.

## Conclusions

In conclusion, our experience with 144 cases of MICS demonstrates the feasibility and advantages of employing minimally invasive techniques in a diverse patient population. The balance of procedural diversity, combined with favorable clinical outcomes, highlights the potential for MICS to become a standard practice in cardiothoracic surgery even in the developing limited resources like Peshawar, Pakistan. Future studies should continue to evaluate long-term outcomes, the impact of various comorbidities, and the efficacy of newer techniques to further refine our approach to MICS.
